# Exit Interviews Exploring Patients’ Experience of Change in Crohn’s Disease Symptoms During the Mirikizumab Phase 3 Clinical Trial In Adult Patients With Moderately-to-Severely Crohn’s Disease

**DOI:** 10.1093/crocol/otae079

**Published:** 2024-12-16

**Authors:** Theresa Hunter Gibble, Jake Macey, Harriet Makin, Rodica Rosu, Katie Mellor, Helen Kitchen, Emily Hon, Marla Dubinsky

**Affiliations:** Medical Affairs, Eli Lilly and Company, 893 Delaware St., Indianapolis, IN, USA; Clinical Outcomes Assessment, Clarivate, 70 St. Mary Axe, London, UK; Clinical Outcomes Assessment, Clarivate, 70 St. Mary Axe, London, UK; Clinical Development, Eli Lilly and Company, 893 Delaware St., Indianapolis, IN, USA; Clinical Outcomes Assessment, Clarivate, 70 St. Mary Axe, London, UK; Clinical Outcomes Assessment, Clarivate, 70 St. Mary Axe, London, UK; Clinical Development, Eli Lilly and Company, 893 Delaware St., Indianapolis, IN, USA; Icahn School of Medicine at Mount Sinai, 1 Gustave L. Levy Place, New York, IN, USA

**Keywords:** qualitative interviews, patient-report outcomes, clinical outcomes assessment, meaningful change, Crohn’s disease

## Abstract

**Background:**

Exit interviews with patients who completed the Phase 3 VIVID-1 mirikizumab clinical trial for moderately-to-severely active Crohn’s disease explored the content validity of bowel urgency, stool frequency, and abdominal pain patient-reported outcome measures and perceptions of meaningful within-patient change and remission in these key Crohn’s disease symptoms.

**Methodology:**

Cognitive debriefing explored patient understanding of the bowel urgency numeric rating scale (Urgency NRS), Crohn’s Disease Activity Index: Stool Frequency (CDAI-SF) and Abdominal Pain (CDAI-AP), and patient global rating/impression of severity/change (PGRS/PGIC). Perceptions of meaningful change and remission were explored qualitatively. Transcripts were analyzed using directed content and framework analysis.

**Results:**

Interviewed participants (*N* = 62; mean age 44.8 years, 55% female, mean 12.0 years since Crohn’s disease diagnosis) were from the United States (*n* = 29), Czech Republic (*n* = 10), Poland (*n* = 8), Germany (*n* = 7), Canada (*n* = 4), Australia (*n* = 3), and the United Kingdom (*n* = 1). Participants understood the Urgency NRS, CDAI-SF, and CDAI-AP and could use them to rate their bowel urgency, stool frequency, and abdominal pain. Participants considered these symptoms when responding to the PGRS/PGIC. Meaningful change was described as symptom relief resulting in the ability to live daily life without pain or fear/need of rushing to the toilet. Most participants agreed with a proposed remission definition of ≤3 type 6/7 bowel movements and None/Mild abdominal pain.

**Discussion:**

The Urgency NRS, CDAI-SF, and CDAI-AP are content-valid patient-reported outcome measures in Crohn’s disease. The PGRS/PGIC are conceptually related global assessments of bowel urgency, stool frequency, and abdominal pain. Patients considered reduction in these symptoms as meaningful and remission.

## Introduction

Crohn’s disease is an inflammatory bowel disease with rising incidence rates estimated at 0–20.2 cases per 100 000 person-years in North America and 0.3–12.7 cases per 100 000 person-years in Europe.^1–3^ Symptoms include abdominal pain, urgent and frequent bowel movements, and liquid/soft stools, which are bothersome to patients and affect their physical and emotional well-being and daily functioning.^4,5^

In Phase 2 (SERENITY) and Phase 3 (VIVID-1) clinical trials, the safety and efficacy of Mirikizumab were demonstrated in patients with moderately-to-severely Crohn’s disease.^6–8^ VIVID-1 was a 52-week, Phase 3, multicenter, randomized, double-blind clinical trial (NCT03926130) that evaluated the safety and efficacy of mirikizumab compared to placebo in patients with moderately-to-severely active Crohn’s disease. VIVID-1 included several patient-reported outcome measures to assess key Crohn’s disease symptoms, including a bowel urgency numeric rating scale (NRS)^9^ and single items assessing stool frequency and abdominal pain from the Crohn’s Disease Activity Index (CDAI).^10,11^

Regulatory and best practice guidance from the Food and Drug Administration and health economics and outcomes research professionals detail the importance of and requirements for ensuring that patient-reported outcome measures used in clinical trials are fit-for-purpose in the context of use and population of interest.^12–18^ This includes establishing that the patient-reported outcome measure is well understood and interpreted as comprehensively assessing the intended concept of interest (content validity) and that score differences on the measure are meaningful and reflect the expected differences in patients’ experience of the concept of interest. Clinical trial exit interviews are ideal for exploring the content validity of patient-reported outcome measures directly in the context of use and population of interest.^19^ Furthermore, exploring meaningfulness of patient-reported outcome score changes with patients involved in a clinical trial allows patients to comment on actual change (or otherwise) in the same outcomes as those evaluated by the clinical trial endpoints, providing additional information to contextualize endpoints. For this reason, clinical trial exit interviews to explore meaningful changes qualitatively and in patient-reported outcome scores have become increasingly common in recent years.^20–24^ Similarly, exploring perceptions of remission in a sample in which some are likely to have achieved remission could support predefined remission definitions. Clinical trial participants are the most relevant sample of patients to inform the remission definition that relates to their treatment.

This study comprised exit interviews conducted with adult patients with moderately-to-severely active Crohn’s disease who had completed the 52-week VIVID-1 clinical trial period to explore the content validity of bowel urgency, stool frequency, and abdominal pain patient-reported outcome measures and to understand patient experiences of change and perceptions of meaningful within-patient change and remission in these key Crohn’s disease symptoms.

## Materials and Methods

### Sample

VIVID-1 trial participants who were actively participating in the trial upon commencement of the interview study and could provide written informed consent to participate in a 90-min interview within 3 weeks of week 52, having completed all clinical trial treatment period procedures were eligible (trial arm/response was blinded). Participants were required to have internet and computer/tablet access to enable the interviewer to share materials onscreen during the interview (access to/participation via smartphone only was not permitted because of insufficient screen size to view materials).

At least 40 exit interviews were targeted as this sample size was hypothesized to be sufficient to determine the content validity of the patient-reported outcome measures and understand patients’ perceptions of clinically meaningful change based on guidance,^17,25^ past experience,^26,27^ and other similar studies.^28^ This sample size was also hypothesized to provide some sample characteristic diversity; however, no specific diversity targets were sought (eg, sex, location, education level).

### Procedures

A total of 34 mirikizumab clinical trial sites across the United States (17 sites), Poland (5 sites), Czech Republic (4 sites), Australia (3 sites), Germany (3 sites), Canada (1 site), and the United Kingdom (1 site) approached VIVID-1 participants about the exit interview study. Trial site staff obtained written informed consent for exit interview study participation from eligible and interested participants and completed an exit interview study case report form, which collected sample characteristics.

Interviews within 3 weeks of participants’ final trial (Visit 17/week 52) were targeted to minimize chance of recall bias. Interviews were conducted remotely using online communication software (eg, Microsoft Teams) by professional, experienced research interviewers who were trained on the study methods and interview guide, separate from the clinical trial team, in the participant’s local language and lasted approximately 90 min. Participants were compensated for their time and participation in accordance with local laws and regulations.

### Materials

Interviews were conducted according to a semi-structured exit interview guide, developed by clinical outcome assessments researchers experienced in qualitative interview studies (authors J.M., H.K., H.M.) with input from the study sponsor, which included questions to qualitatively explore change experienced during VIVID-1, cognitively debrief the patient-reported outcome measures, and understand experienced and meaningful change and remission in relation to first and last visit scores on the patient-reported outcome measures (Table S1). The following key symptom patient-reported outcome measures were shared onscreen during relevant sections of the interview to facilitate understanding (participants who had difficulty with the online communication software were sent the PowerPoint presentation by email (this was recorded as a minor protocol deviation)):

- Urgency numeric rating scale (Urgency NRS)—Single item to assess severity of bowel urgency in the past 24 hours (“How severe was your urgency (sudden or immediate need) to have a bowel movement during the past 24 hours?”) on an 11-point NRS anchored from 0 “No urgency” to 10 “Worst possible urgency.”
- Crohn’s Disease Activity Index: Stool Frequency (CDAI-SF) item—Single item from the CDAI that provides Bristol stool chart and asks participants to report liquid or very soft stool frequency in the past 24 hours (“Of your total bowel movements in the past 24 hours, how many were liquid or very soft (Type 6 or 7)?”).
- Crohn’s Disease Activity Index: Abdominal Pain (CDAI-AP) item—Single item from the CDAI to assess worst abdominal pain in the past 24 hours (“Please think about your worst pain experienced in the past 24 hours. How would you rate your abdominal pain over the past 24 hours?”) using a 4-point scale 0 “None” to 3 “Severe.”
- Patient Global Rating of Severity (PGRS)—Single items to assess Crohn’s disease severity in the past 24 hours (“How would you rate your overall Crohn’s Disease symptoms over the past 24 hours?”) on a 6-point Likert scale from 0 “None” to 5 “Very severe” and
- Patient Global Impression of Change (PGIC)—Single item to assess change during the clinical trial (“Select the response below that best describes how your Crohn’s Disease symptoms are now, compared with how they were before you started taking this medicine”) on an 8-point Likert scale from 0 “Very much better” to 7 “Very much worse.”

### Analysis

Interview recordings were transcribed verbatim in the local language and non-English transcripts were then translated into English for analysis. Analysis was guided by a brief qualitative analysis plan and transcripts were coded and analyzed using ATLAS.ti v9. Trial blinding was maintained during all analyses.

Directed qualitative content analysis^29–31^ techniques were used to iteratively identify and apply domains and concepts that enabled an understanding of participants’ individual perspectives and experiences of symptoms, impacts, and meaningful change. Framework analysis^32^ with specific, pre-defined codes was applied to patient-reported outcome measure cognitive testing questions to explore participant understanding, relevance, and interpretation of the instructions, response options, and recall period. Approximate thresholds of meaningful change were estimated based on reporting by a majority of the sample (>50%).

Sample characteristics data were analyzed using Microsoft Excel and reported descriptively (eg, mean and standard deviation, frequency, and percentage).

## Ethical Considerations

This exit interview study was submitted and approved as an addendum to the VIVID-1 clinical trial protocol by local ethics committees/institutional review boards in accordance with local regulations. Participation was optional for both trial sites and clinical trial participants. Written informed consent to participate was obtained from participants by the trial site staff, which included consent to provide information and data necessary for exit interview study conduct to the exit interview study team. Additional verbal consent to participate and be recorded was obtained from participants by the interviewer at the time of the interview. Unique identifiers (IDs) were assigned to participants to protect their identities. Unique identifiers comprised a number (01-62) followed by a country code provided ≥5 participants from the country were included. Numbers were assigned based on order in which interviews were scheduled (which may have differed to the order of interview conduct). County codes were used for Germany (DE), the United States of America (US), Poland (PL), and Czech Republic (CZ) but not Australia, Canada, and the United Kingdom of Great Britain to preserve participant anonymity as there were fewer than 5 participants from each of these countries). In addition, adverse events spontaneously described by participants during their interviews were reported to clinical sites and documented in line with the clinical trial protocol.

## Results

### Sample characteristics

A total of 62 VIVID-1 trial participants from the United States (*n* = 29, 47%), Czech Republic (*n* = 10, 16%), Poland (*n* = 8, 13%), Germany (*n* = 7, 11%), Canada (*n* = 4, 6%), Australia (*n* = 3, 5%), and the United Kingdom (*n* = 1, 2%) completed an exit interview.

A median of 55.2 weeks (range 51.3–65.0 weeks) had elapsed between baseline and final VIVID-1 trial visits, and exit interviews were conducted a median of 1.9 weeks (range 0.0–3.7 weeks) after final VIVID-1 trial visits. However, three interviews were conducted 1 (*n* = 2) and 5 (*n* = 1) days outside of this window to accommodate participant availability (these were recorded as minor protocol deviations). No differences in participant responses or recall ability were observed in the three interviews conducted outside of the target interview window (≤3 weeks after final VIVID-1 trial visit) compared to those conducted within the interview window.

Participants were diagnosed with Crohn’s disease for a median of 7.9 years (range 1.4-54.1 years), were a median of 44.0 years of age (range 22.0-75.0 years) and most were White (*n* = 56/62, 90%) and not of Hispanic or Latino origin (*n* = 47/60, 78%). Just over half the sample were female (*n* = 34/62, 55%) and/or employed full time (*n* = 32/60, 53%). Participants’ highest education levels were high school diploma or equivalent (*n* = 26/59, 44%), university or post-graduate degree (*n* = 15/59, 25%), college degree, apprenticeship or professional qualification (*n* = 13/59, 22%) and primary education or high school without diploma (*n* = 5/59, 8%). See Table 1 for full sample characteristics by country.

**Table 1.** Sample characteristics.

Characteristic	US	CZ	PL	DE	CA/AU/UK	Total
Years since diagnosis^a^*(N)*	*28/29*	*10/10*	*8/8*	*7/7*	*8/8*	*61/62*
Mean (SD)	13.1 (14.2)	12.0 (8.4)	12.7 (8.4)	5.9 (3.7)	13.1 (14.9)	12.0 (11.9)
Range	1.5-54.1	2.1-26.1	1.8-26.1	1.7-11.6	1.4-36.1	1.4-54.1
Age *(N)*	*29/29*	*10/10*	*7/8*	*7/7*	*8/8*	*61/62*
Mean (SD)	48.1 (16.7)	43.7 (11.7)	36.4 (9.7)	34.0 (7.5)	51.3 (11.0)	44.8 (14.6)
Range	24.0-75.0	27.0-61.0	23.0-48.0	22.0-45.0	31.0-61.0	22.0-75.0
Sex *(N)*	*29/29*	*10/10*	*8/8*	*7/7*	*8/8*	*62/62*
Female	16 (55%)	7 (70%)	4 (50%)	2 (29%)	5 (63%)	34 (55%)
Male	13 (45%)	3 (30%)	4 (50%)	5 (71%)	3 (38%)	28 (45%)
Race (*N)*	*29/29*	*10/10*	*8/8*	*7/7*	*8/8*	*62/62*
White	26 (90%)	9 (90%)	8 (100%)	7 (100%)	6 (75%)	56 (90%)
Black or African American	2 (7%)	0 (0%)	0 (0%)	0 (0%)	0 (0%)	2 (3%)
Asian	1 (3%)	0 (0%)	0 (0%)	0 (0%)	0 (0%)	1 (2%)
Other	0 (0%)	1 (10%)	0 (0%)	0 (0%)	1 (13%)	2 (3%)
Prefer not to say	0 (0%)	0 (0%)	0 (0%)	0 (0%)	1 (13%)	1 (2%)
Hispanic/Latino origin *(N)*	*29/29*	*10/10*	*8/8*	*5/7*	*8/8*	*60/62*
Not Hispanic or Latino	28 (97%)	10 (100%)	8 (100%)	5 (100%)	8 (100%)	59 (98%)
Hispanic or Latino	1 (3%)	0 (0%)	0 (0%)	0 (0%)	0 (0%)	1 (2%)
Work status *(N)*	*29/29*	*10/10*	*8/8*	*5/7*	*8/8*	*60/62*
Employed full-time	15 (52%)	5 (50%)	2 (25%)	4 (80%)	6 (75%)	32 (53%)
Retired	5 (17%)	1 (10%)	0 (0%)	0 (0%)	0 (0%)	6 (10%)
Unemployed	5 (17%)	0 (0%)	0 (0%)	0 (0%)	1 (13%)	6 (10%)
Employed part-time	2 (7%)	2 (20%)	0 (0%)	1 (20%)	1 (13%)	6 (10%)
Homemaker	1 (3%)	1 (10%)	0 (0%)	0 (0%)	0 (0%)	2 (3%)
Student	0 (0%)	0 (0%)	2 (25%)	0 (0%)	0 (0%)	2 (3%)
Paid sick leave	0 (0%)	1 (10%)	1 (13%)	0 (0%)	0 (0%)	2 (3%)
Job order contract	0 (0%)	0 (0%)	1 (13%)	0 (0%)	0 (0%)	1 (2%)
Own business	0 (0%)	0 (0%)	1 (13%)	0 (0%)	0 (0%)	1 (2%)
Pension	0 (0%)	0 (0%)	1 (13%)	0 (0%)	0 (0%)	1 (2%)
Other (not specified)	1 (3%)	0 (0%)	0 (0%)	0 (0%)	0 (0%)	1 (2%)
Education level *(N)*	*29/29*	*10/10*	*8/8*	*5/7*	*7/8*	*59/62*
High/secondary school diploma or equivalent	11 (38%)	5 (50%)	3 (38%)	4 (80%)	3 (43%)	26 (44%)
College or associate degree or equivalent	9 (31%)	0 (0%)	0 (0%)	0 (0%)	2 (29%)	11 (19%)
Graduate/post-graduate degree	2 (7%)	0 (0%)	5 (63%)	1 (20%)	2 (29%)	10 (17%)
Bachelor’s degree	5 (17%)	0 (0%)	0 (0%)	0 (0%)	0 (0%)	5 (8%)
High/secondary school without diploma	0 (0%)	3 (30%)	0 (0%)	0 (0%)	0 (0%)	3 (5%)
Primary education only	0 (0%)	2 (20%)	0 (0%)	0 (0%)	0 (0%)	2 (3%)
Medical Assistant Certification	1 (3%)	0 (0%)	0 (0%)	0 (0%)	0 (0%)	1 (2%)
Nursing (RN) Diploma Program (3 years)	1 (3%)	0 (0%)	0 (0%)	0 (0%)	0 (0%)	1 (2%)

“N” indicates missing data.

DE = Germany, US = United States of America, PL = Poland, CZ = Czech Republic, AU = Australia, CA = Canada, UK = United Kingdom of Great Britain.

CA/AU/UK combined because of small samples sizes (*N* < 5 each).

^a^16 CRFs detailed year of diagnosis only. Where month was not provided, “years” between diagnosis and interview was used.

### Cognitive Debriefing of Key Symptom Patient-Reported Outcome Measures

#### Urgency NRS

Most participants (*n* = 57/62) demonstrated a clear understanding of the Urgency NRS. Responses included consideration of how actual experiences of bowel urgency compared to what was perceived to be “normal,” *“how quickly” (04)* bathroom access was needed, and the ability to control or hold bowel movements. The response scale was well understood and represented increasing bowel urgency ranging from No urgency [0] to an immediate, sudden need or loss of bowel control [Worst urgency, 10]. Two participants noted difficulty with the 24-hour recall period related to variation in their experience of bowel urgency.

#### CDAI-SF

All participants (*n* = 31/31) who reviewed the CDAI-SF understood the Bristol Stool Chart stool types included in the CDAI-SF. Ten participants initially described considering the type of stool, rather than the number of type 6 or type 7 stools when answering the CDAI-SF, however, most of these participants (*n* = 7) were able to provide correct responses and/or recalled their trial responses following clarification from the interviewer or reading the question again. Ultimately, most participants (*n* = 28/31) clearly understood that the item required them to enter the number of type 6 or 7 bowel movements they had in the past 24 hours.

#### CDAI-AP

All participants (*n* = 30/30) understood that the CDAI-AP was asking them to rate their level of abdominal pain and were able to select a response. Participants generally understood (*n* = 17/20) that abdominal pain referred to pain around their abdomen, tummy, belly, stomach, or intestines and described the pain using terms such as cramping, stabbing, discomfort, and hurt.

#### Patient Global Rating of Severity and Patient Global Impression of Change

All participants (*n* = 62/62) reviewed and overall understood the PGRS and PGIC global ratings of Crohn’s disease. Some participants (*n* = 11) reported problems with using the PGRS response options related to difficulty choosing a category (eg, subjectivity, temporal fluctuations in symptom experience) and/or difficulty with distinguishing between categories that were considered similar (eg, Very Mild and Mild). Two participants were unsure about how to interpret two different PGIC response options (*Much better* and *A little better*). Ultimately, all participants could use the global items, and most, spontaneously, or when probed, thought about key Crohn’s disease symptoms when providing a response (Table 2).

**Table 2.** Symptoms considered in participants’ patient global impression of change and patient global impression of change responses.

Symptom	PGRS, *n* (%)		PGIC, *n* (%)	
	Considered^a^	Not considered	Considered^a^	Not considered
Abdominal pain	59 (98%)	1 (2%)	51 (96%)	2 (4%)
Frequency of bowel movements	45 (88%)	6 (12%)	51 (100%)	0 (0%)
Water/liquid or very soft stools	49 (91%)	5 (9%)	46 (90%)	5 (10%)
Fatigue	43 (73%)	16 (27%)	36 (72%)	14 (28%)
Urgency of bowel movements	42 (82%)	9 (18%)	32 (78%)	9 (22%)

^a^Reported spontaneously or when probed.

### Meaningful Change in Symptoms During VIVID-1

#### Bowel urgency on the Urgency NRS

Most (*n* = 55/62) participants reported improvement in Urgency NRS score during the clinical trial (Table 3). Almost all (*n* = 51/55) felt the improvement they experienced was meaningful because “I could start living again. […] having a normal life as much as possible.” (04) and “I can feel, sort of, more confident that I don’t need to be next to the toilet at every moment all the time.” (18-PL).

**Table 3.** Self-reported meaningful change in bowel urgency on the Urgency numeric rating scale.

Change	Experienced change (*N* = 62), *n* (%)^a^			Smallest meaningful improvement (*N* = 54), *n* (%)
	Meaningful	Not meaningful	Meaningfulness unclear/missing	
10-Point improvement	3 (5%)	0 (0%)	0 (0%)	–
9-Point improvement	3 (5%)	0 (0%)	0 (0%)	–
8- or 9-Point improvement	1 (2%)	0 (0%)	0 (0%)	–
8-Point improvement	1 (2%)	0 (0%)	1 (2%)	–
7-Point improvement	5 (8%)	0 (0%)	0 (0%)	2 (4%)
6-Point improvement	8 (13%)	0 (0%)	0 (0%)	–
5 to 6-Point improvement	1 (2%)	0 (0%)	0 (0%)	–
5-Point improvement	10 (16%)	0 (0%)	0 (0%)	5 (9%)
4 to 5-Point improvement	1 (2%)	0 (0%)	0 (0%)	–
4-Point improvement	2 (3%)	0 (0%)	0 (0%)	6 (11%)
3 or 4-Point improvement	–	–	–	1 (2%)
3-Point improvement	14 (23%)	0 (0%)	0 (0%)	17 (31%)
2 or 3-Point improvement	–	–	–	1 (2%)
2-Point improvement	0 (0%)	3 (5%)	0 (0%)	12 (22%)
1 or 2-Point improvement	1 (2%)	0 (0%)	0 (0%)	–
1-Point improvement	1 (2%)	0 (0%)	0 (0%)	10 (19%)
0-Point no change	1 (2%)	0 (0%)	3 (5%)	–
1-Point worsening	0 (0%)	0 (0%)	1 (2%)	–
2-Point worsening	0 (0%)	1 (2%)	0 (0%)	–
4-Point worsening	1 (2%)	0 (0%)	0 (0%)	–

^a^Relates to change from “first trial visit before taking the study medication” to “last trial visit” (*n* = 55) or “time of interview” (*n* = 7).

“–” indicates change not reported in corresponding interview question/task.

A 3-point Urgency NRS improvement was identified as the smallest meaningful change (*n* = 40/54; Table 3). Participants reported this change would result in reduced bathroom frequency, less worry, feeling safer and calmer and mean they could leave the house more easily, go to work, and be able to travel without taking breaks. The ability to complete more activities would result in improved mood.

#### Liquid/very soft (type 6/7) stool frequency on the CDAI-SF

The number of type 6/7 bowel movements participants recalled having, to the best of their ability, at the time of their first trial visit varied considerably, ranging from 1 to 40 bowel movements (*n* = 19/30 reported ≥ 6 movements). Almost all participants (*n* = 28/30) experienced an improvement in frequency of type 6/7 bowel movements during the clinical trial, which similarly varied (Table 4). However, all considered their individual improvement meaningful because “that I can function normally. I can go shopping or work or whatever else needs to happen without having to worry about constantly needing to use the bathroom wherever I go.” (08-US), “your depression would start lifting, you would have more energy, because you could go do things, and you’re not trapped in the house” (44-US) and “I do not have any problems with my underwear being soiled. I perhaps have the problem that I need to hurry to the toilet, but the consistency is not so loose, so I can hold it in.” (30-CZ).

**Table 4.** Self-reported meaningful change in stool frequency on the CDAI-SF.

Change in bowel movement frequency	Experienced change (*N* = 30), *n* (%)		Smallest meaningful improvement (*N* = 22), *n* (%)
	Meaningful	Not meaningful	
40 fewer movements	1 (3%)	0 (0%)	–
17 fewer movements	1 (3%)	0 (0%)	–
14 to 17 fewer movements	1 (3%)	0 (0%)	1 (5%)
14 fewer movements	1 (3%)	0 (0%)	–
12 fewer movements	1 (3%)	0 (0%)	–
10 to 15 fewer movements	1 (3%)	0 (0%)	–
10 fewer movements	1 (3%)	0 (0%)	2 (9%)
8 fewer movements	1 (3%)	0 (0%)	–
7 fewer movements	1 (3%)	0 (0%)	–
6 fewer movements	4 (13%)	0 (0%)	1 (5%)
5 to 6 fewer movements	–	–	1 (5%)
5 fewer movements	3 (10%)	0 (0%)	2 (9%)
4 to 5 fewer movements	1 (3%)	0 (0%)	1 (5%)
4 fewer movements	5 (17%)	0 (0%)	1 (5%)
3 fewer movements	3 (10%)	0 (0%)	2 (9%)
2 to 6 fewer movements	–	–	1 (5%)
2 to 3 fewer movements	–	–	3 (14%)
2 fewer movements	1 (3%)	0 (0%)	3 (14%)
1 fewer movement	2 (7%)	0 (0%)	4 (18%)
0 (no change)	1 (3%)	0 (0%)	–
13 more movements	1 (3%)	0 (0%)	–

“–” indicates change not reported in corresponding interview question/task.

In addition, participants (*n* = 12/22) considered one to 3 fewer type 6/7 bowel movements per day as the smallest meaningful change (Table 4). Some participants (*n* = 6) commented that their desired improvement would allow them to be more functional, enjoy their lives and/or feel generally better.

#### Abdominal pain on the CDAI-AP

Most participants (*n* = 25/29) reported experiencing improvement in abdominal pain on the CDAI-AP during VIVID-1 (Table 5). Most of the participants who experienced a 1-point change (*n* = 4/6) did not consider this to be meaningful but other participants asked (*n* = 20/24) felt their improvement was meaningful because “I was basically able to do everything I could do with abdominal pain, just with a lot more energy and concentration because I didn’t have any pain.” (40-DE), “now I can easily handle the cooking, cleaning, working in the garden, and when I need to go into town, now I go there” (58-CZ) and “the comfort of not having to think about what I eat. And […] functioning is different after all. I have kind of started to move now, I do sports. I don’t feel anxious that my abdomen aches” (34-PL).

**Table 5.** Self-reported meaningful change in abdominal pain on the CDAI-AP.

Change	Experienced change (*N* = 29), *n* (%)			Smallest meaningful improvement (*N* = 23), *n* (%)
	Meaningful	Not meaningful	Meaningfulness missing	
3-Point improvement	4 (14%)	0 (0%)	0 (0%)	–
2- to 3-Point improvement	3 (10%)	0 (0%)	0 (0%)	–
2-Point improvement	9 (31%)	0 (0%)	1 (3%)	–
1- to 3-Point improvement	2 (7%)	0 (0%)	0 (0%)	4 (17%)
1-Point improvement	2 (7%)	4 (14%)	0 (0%)	19 (83%)
0-Point (no change)	1 (3%)	0 (0%)	3 (10%)	–

“–” indicates change not reported in corresponding interview question/task.

Participants (*n* = 19/26) reported that a 1-point improvement in the CDAI-AP would be the smallest meaningful change (Table 5), generally, because they would be less affected by their abdominal pain and mostly able to carry on with their daily activities.

#### Patient Global Rating of Severity and Patient Global Impression of Change

Most participants reported a 1-point to 5-point improvement (*n* = 59/62) in their PGRS response between the first and the last visit and achieving Much better or Very much better (*n* = 48/62) on the PGIC (Table 6). The majority of participants reported their improvement was meaningful. Almost two-thirds of participants asked (*n* = 32/49), reported that a 1-point PGRS improvement would be the smallest acceptable meaningful change as they would experience reduced symptoms, limiting the impact of their symptoms on their work life and reducing the number of hospitalizations that were experienced. In addition, participants could relate their first and last visit responses and change in Urgency NRS (Figure S1) and stool frequency (Figure S2) to the PGRS and PGIC categories.

**Table 6.** Self-reported meaningful change on the patient global rating of severity and patient global impression of change.

Change	Experienced change (*N* = 62), *n* (%)			Smallest meaningful improvement (*N* = 49), *n* (%)
	Meaningful	Not meaningful	Meaningfulness missing	
*PGRS*				
5-Point improvement	3 (5%)	0 (0%)	0 (0%)	–
4-Point improvement	7 (11%)	0 (0%)	1 (2%)	–
3-Point improvement	11 (18%)	0 (0%)	0 (0%)	1 (2%)
*2-Point to 3-point improvement*	*0 (0%)*	*1 (2%)*	*1 (2%)*	*–*
2-Point improvement	21 (34%)	0 (0%)	0 (0%)	15 (31%)
*1-Point to 2-point improvement*	*1 (2%)*	*0 (0%)*	*0 (0%)*	*1 (2%)*
1-Point improvement	10 (16%)	2 (3%)	0 (0%)	32 (65%)
*No change to 1-point improvement*	*0 (0%)*	*0 (0%)*	*1 (2%)*	–
No change	0 (0%)	0 (0%)	2 (3%)	–
1-Point worsening	0 (0%)	0 (0%)	1 (2%)	–
*PGIC*				–
Very much better	17 (27%)	0 (0%)	3 (5%)	–
*Much better to very much better*	*1 (2%)*	*0 (0%)*	*0 (0%)*	–
Much better	26 (42%)	0 (0%)	1 (42%)	–
A little better	5 (8%)	3 (5%)	1 (2%)	–
No change	1 (2%)	2 (3%)	1 (2%)	–
Much worse	0 (0%)	0 (0%)	1 (3%)	–

“–” indicates change not reported in corresponding interview question/task (smallest meaningful change on the PGIC was not explored).

### Remission

#### Bowel urgency

Bowel urgency remission on the Urgency NRS was defined by participants as a score of two or less (*n* = 41/57). Participants defined bowel urgency remission as reduced or absent bowel urgency and that remission meant having more time to find the bathroom or not needing to stop what they were doing to find the bathroom.

#### Liquid/very soft (type 6/7) stool frequency

Just over a third of participants asked (*n* = 11/29) considered only zero type 6/7 bowel movements in a typical 24 hours would indicate their Crohn’s disease was in remission. Other participants (*n* = 18/29) considered bowel movement frequencies from “0-1” to “4-5” as remission. Participants (*n* = 10) reported perceived positive impacts of remission included feeling happier and generally better or normal, being able to function, travel, socialize, and play with their children, and less worry about their disease or bathroom locations.

#### Abdominal pain

Nearly half of participants (*n* = 12/26) reported that they would consider their Crohn’s disease to be in remission if they were in the CDAI-AP response category *None* on most days. Other participants reported *Mild* (*n* = 9/26), *None* to *Mild* (*n* = 4/26), or described “no abdominal pain” (10-US) as remission. Participants (*n* = 8) described positive impacts of remission as feeling that their pain was subsiding, feeling positive or normal, being able to stop taking medicine every day and not feeling like they need to curl up due to the pain.

#### Overall remission definition

Participants (*n* = 27) were asked if they would consider having three or fewer type 6/7 bowel movements on the CDAI-SF and abdominal pain at *Mild* or *None* on the CDAI-AP in 24 hours on most days as “in remission.” Most participants (*n* = 20/27) reported that they would consider this to be remission *because* “I had my life back. And just that positivity was able to come back. Like, wow, this has improved this much. It just makes you positive, and it just makes you grateful.” (48-US) and “I wouldn’t have to go to the toilet that often, yeah. That the diarrhea is very tiring. So, when there’s less diarrhea […] I feel better overall.” (58-CZ). Other participants (*n* = 7/27) reported they would not consider this as remission. These participants needed no abdominal pain (*n* = 3/7), two or fewer (*n* = 2/7), or zero (*n* = 2/7) type 6/7 bowel movements for remission.

Most participants (*n* = 18/25) reported that they considered their Crohn’s disease as “in remission” at the end of the clinical trial because they had “no symptomology” (02-US) or their symptoms had reduced. Other participants (*n* = 7/25) reported that they did not consider their Crohn’s disease as “in remission” at the end of the clinical trial because of limited change in the frequency of type 6/7 bowel movements, presence of abdominal pain and/or no change or presence of Crohn’s disease symptoms.

## Discussion

The VIVID-1 clinical trial exit interviews confirmed the content validity of key Crohn’s disease symptom patient-reported outcome measures and informed an understanding of patient experiences and perceptions of meaningful within-patient change and remission in key symptoms.

Strong participant understanding and appropriate use of the Urgency NRS, CDAI-SF, CDAI-AP, PGRS, and PGIC were demonstrated in the exit interviews, supporting the content validity of these patient-reported outcome measures. The Urgency NRS was originally developed in ulcerative colitis.^9^ This study’s results support existing evidence of its content validity in Crohn’s disease.^33^ To date, content validity of the CDAI has been largely assumed by quantitative evidence of its responsiveness to clinical severity changes and its wide use in clinical trials;^34^ however, this does not align with current best practice standards for qualitative evidence of content validity.^12–18^ This study provides the first qualitative evidence of content validity for the CDAI-SF and CDAI-AP items. The majority of participants considered the symptoms of bowel urgency, stool frequency, and abdominal pain when providing a rating to the PGRS and PGIC, suggesting these global items are suitable anchors conceptually for the key symptom patient-reported outcome measures in VIVID-1. Indeed, moderate (CDAI-SF) and large (Urgency NRS, CDAI-AP) correlations between these measures and the PGRS have been identified in analysis of their psychometric properties.^9,35^

Most interviewed participants reported experiencing improvement in the symptoms of bowel urgency, type 6/7 stool frequency, and abdominal pain to varying degrees. Although it is unknown into which trial treatment arm participants were enrolled, most reported the improvements to be meaningful as they were freed from the burden of Crohn’s disease and able to complete their daily activities, socialize with friends and family, exercise and work without pain or fear of rushing to find a toilet or soiling themselves. These qualitative reports of meaningful change can inform understanding of quantitatively derived change thresholds.

Remission in Crohn’s disease bowel urgency was defined by participants as an Urgency NRS score of ≤2. When considering stool frequency and abdominal pain separately, remission was defined as ≤1 type 6/7 bowel movements and None or Mild abdominal pain. Most participants agreed that a proposed remission definition of ≤3 type 6/7 bowel movements and None or Mild abdominal pain was appropriate. Seventeen participants who self-reported achieving this remission definition at the end of the trial considered their Crohn’s disease as “in remission”; one additional participant with 6 type 6/7 bowel movements and Mild abdominal pain also reported achieving remission.^35^ However, the self-reported interview study results were not matched to trial data to confirm whether these patients achieved the remission definition.

Overall, the exit interview results support previous qualitative research that has identified abdominal pain and urgent, frequent loose stools as burdensome and the most bothersome Crohn’s disease symptoms that would cause patients to seek treatment.^4,5,33^ Descriptions of change in symptoms and impacts experienced across VIVID-1 were consistent with symptom and impact descriptions in these previous qualitative studies. The remission thresholds identified align with previously reported patient definitions of remission, including watery/liquid bowel movement frequency and abdominal pain, and suggest that patients consider “remission” as experiencing reduced or less severe, not necessarily absent, symptoms.^4,5^

Limitations of this study are acknowledged. Patients who discontinued were not interviewed and these patients may have experienced worse outcomes compared to patients who completed VIVID-1. A high proportion of participants reported improvement in their Crohn’s disease symptoms. This could indicate either mirikizumab or its comparator (ustekinumab) are successful treatments for Crohn’s disease, there was a strong placebo effect present or participants with positive trial experiences who experienced an improvement were more inclined or physically able to participate in a 90-min interview. The latter is hypothesized to be the most likely explanation for this finding, but trial blinding was maintained during analysis and, ultimately, confirmation of the success of mirikizumab in treating Crohn’s disease is subject to analysis of clinical trial data. While the sample size of 62 is relatively large for a qualitative study, the interview content was divided across the sample to ensure that data relating to all study objectives could be collected within the limited interview time. Racial and ethnic diversity was low in this study; however, consistency between interviews suggests that further interviews are unlikely to provide new or more information. The sample was sufficiently diverse across other characteristics including educational background, age, and sex, and was geographically diverse including North America, eastern Europe, western Europe and, to a lesser degree, Australasia. English transcripts were analyzed so nuances in terminology and differences in understanding may have been lost in translation. However, this was somewhat mitigated by “gold standard” translation in which native language verbatim transcripts were created and then translated into English (instead of direct to English transcription). In addition, this study was not designed to assess differences between localized versions of the patient-reported outcome measures or regional differences in symptom experience and formal subgroup analysis by region was not undertaken.

## Conclusion

The Urgency NRS, CDAI-SF, and CDAI-AP are content-valid patient-reported outcome measures of bowel urgency, type 6/7 stool frequency, and abdominal pain. The PGRS and PGIC are well understood and conceptually related to these patient-reported outcome measures and therefore likely to be suitable as anchors for estimating meaningful change. The improvement in these key Crohn’s disease symptoms experienced by VIVID-1 participants was considered meaningful because of the associated improvements to quality of life. Most patients would consider reduction in these symptoms as remission, even if mild symptoms remain.

## Supplementary Material

otae079_suppl_Supplementary_Material

## Data Availability

Data not publicly available.
