# Supplementary material for: Exit Interviews Exploring Patients’ Experience of Change in Crohn’s Disease Symptoms During the Mirikizumab Phase 3 Clinical Trial In Adult Patients With Moderately-to-Severely Crohn’s Disease
Source: Crohns Colitis 360. 2024 Dec 16;7(1):otae079. doi: 10.1093/crocol/otae079 (PMC11744098; doi:10.1093/crocol/otae079)
Supplement: otae079_suppl_Supplementary_Material [file otae079_suppl_supplementary_material.docx]

1. Supplementary material

Supplementary Table 1: Outline of the exit interview guide

| Interview section | Participants^†^ | Description | Example question |
| --- | --- | --- | --- |
| Introduction and warm-up**^‡^** | All | Explanation of the interview procedure, confirmation of written consent to participate, initial questions, about symptoms experienced before the first and at the last trial visit, designed to introduce the participant to the interview | - Thinking about the start of the clinical trial, at the time of your baseline visit on [DD-MONTH-YY], could you tell me about any symptoms you were experiencing? |
| Qualitative exploration of symptoms and treatment administration | All   - PGRS - PGIC - Urgency NRS - Fatigue meaningful change - Injection experience   Even interview IDs   - Stool frequency and CDAI-SF - Abdominal pain and CDAI-AP   Odd interview IDs   - Fatigue and FACIT-F | Open-ended questions to explore the patient experience of a change in stool frequency, abdominal pain, bowel urgency, and fatigue and treatment administration | - I am now going to ask you about urgency of bowel movements. Could you tell me about how urgent your bowel movements were during the clinical trial, thinking about the time period between your first and last visit? - Did you experience any changes in bowel movement urgency during the clinical trial? Can you tell me about that? |
| Cognitive debriefing of patient-reported outcome measures |  | Participants asked to read and complete the patient-reported outcome measures and asked questions to assess their understanding of the patient-reported outcome measure’s instructions, wording, response options, and recall period | - In your own words, what is the question asking you to do? - You selected [answer] for this question, what did you think about when you answered this question? |
| Meaningful change and remission |  | Participants asked about the amount of change they experienced on the patient-reported outcome measures during the trial, about remission in the patient-reported outcome measure concepts, whether they considered the change/remission on the concepts assessed by these patient-reported outcome measures to be a meaningful change, and how they believed the change related to the PGRS and PGIC (except FACIT-Fatigue) | - Thinking about the time between your first visit and last visit, how did your responses to this question change in general?   1. Was this change important or meaningful to you? Why? - Did you feel that the score change from [number] at the first visit of the trial to [number] at the last visit of the trial was a meaningful improvement in bowel urgency? If yes, what did the change mean to you?   1. What difference did the change make to the way you felt?   2. What difference did the change make to the things you were able to do? |
| † To ensure that data relating to all study objectives could be collected within the limited interview time, the content of the interviews was varied depending on whether the participant had been assigned an odd or even interview identifier (ID). ‡ As a result of participant and site feedback, the interview introduction was updated in March 2023 to emphasize that there were no right or wrong answers to the interview questions, that it was OK if participants could not remember their exact patient-reported outcome scores from the trial, acknowledge that some of the interview questions may seem repetitive or obvious but are important for the interviewer to ask and note that the participant’s patience while work through the interview questions is appreciated.  Abbreviations: CDAI-AP = Crohn’s Disease Activity Index – Abdominal Pain; CDAI-SF = Crohn’s Disease Activity Index – Stool Frequency; FACIT-F = Functional Assessment of Chronic Illness Therapy – Fatigue; PGIC = Patient Global Impression of Change; PGRS = Patient Global Rating of Severity. | | | |

Supplementary Figure 1: Urgency NRS scores in relation to the PGRS (N=61) and PGIC (N=56)

| Urgency NRS response option or point change | 0 | 1 | 2 | 3 | 4 | 5 | 6 | 7 | 8 | 9 | 10 |
| --- | --- | --- | --- | --- | --- | --- | --- | --- | --- | --- | --- |
| **Interpretation of Urgency NRS response options in relation to the PGRS response options (n)** | | | | | | | | | | | |
| None | 27 | 22 | 3 | 3 | 1 |  |  |  |  |  |  |
| None to Very mild | 1 |  | 1 | 2 |  |  |  |  |  |  |  |
| Very mild | 1 | 11 | 28 | 17 | 11 | 2 |  | 1 |  |  |  |
| Very mild to Mild | 1 | 2 | 1 | 2 | 3 | 3 | 1 |  |  |  |  |
| Mild |  |  | 4 | 16 | 26 | 18 | 2 |  |  | 1 |  |
| Mild to Moderate |  |  | 1 | 1 | 3 | 3 | 1 |  |  |  |  |
| Moderate |  |  |  | 1 | 1 | 22 | 26 | 14 | 7 | 1 | 2 |
| Moderate to Severe |  |  |  |  |  |  | 5 | 4 | 2 |  |  |
| Severe |  |  |  |  |  | 2 | 3 | 21 | 29 | 11 | 1 |
| Severe to Very severe |  |  |  |  |  |  |  |  |  | 1 | 1 |
| Very severe |  |  |  |  |  |  |  | 1 | 14 | 28 | 42 |
| **Reported Urgency NRS point change in relation to the PGIC response options (n)** | | | | | | | | | | | |
| Very much better^+^ |  |  |  | 3 | 1 | 2 | 5 | 3 | 3 | 2 | 3 |
| Much better^+^ | 1 |  | 1 | 5 | 2 | 3 | 3 | 4 |  |  |  |
| A little better to Much better^+^ |  |  |  |  |  | 1 |  |  |  |  |  |
| A little better^+^ |  | 2 | 3 | 2 | 1 | 2 |  |  |  |  |  |
| No change^+^ | 1 |  | 1 |  |  |  |  |  |  |  |  |
| A little worse^–^ | 1 | 1 | 1 |  |  |  |  |  |  |  |  |
| Much worse^–^ |  |  |  |  | 1 |  |  |  |  |  |  |
| Very much worse^–^ |  |  |  |  | 1 |  |  |  |  |  |  |

+ Change corresponds to improvement (reduction in Urgency NRS score); – Change corresponds to worsening (increase in Urgency NRS score).

Darker tone indicates higher row frequency (n). Frequencies do not equate to sample size as some participants provided a range in their responses.

Supplementary Figure 2: Stool frequency in relation to the PGRS (N=30) and PGIC (N=30)

| Number of type 6 or 7 stools in 24 hours | 0 | 1 | 2 | 3 | 4 | 5 | 6 | 7 | 8 | 9 | 10 | 11 | 12 | 13 | 14 | 15 | 16 | 17 | 18 | 19 | 20 | 40 |
| --- | --- | --- | --- | --- | --- | --- | --- | --- | --- | --- | --- | --- | --- | --- | --- | --- | --- | --- | --- | --- | --- | --- |
| **Interpretation of stool frequency in relation to the PGRS response options (n)** | | | | | | | | | | | | | | | | | | | | | | |
| None | 17 | 5 | 3 | 1 |  |  |  |  |  |  |  |  |  |  |  |  |  |  |  |  |  |  |
| None to Very mild |  | 1 | 2 |  |  |  |  |  |  |  |  |  |  |  |  |  |  |  |  |  |  |  |
| Very mild | 1 | 10 | 9 | 6 | 1 | 1 | 1 | 1 |  |  |  |  |  |  |  |  |  |  |  |  |  |  |
| Very mild to Mild |  |  |  |  |  |  | 1 | 1 | 1 |  |  |  |  |  |  |  |  |  |  |  |  |  |
| Mild | 1 | 4 | 4 | 6 | 6 | 3 | 2 | 2 |  | 1 | 1 |  |  |  |  |  |  |  |  |  |  |  |
| Mild to Moderate |  | 1 | 1 |  |  |  | 1 | 1 |  |  |  |  |  |  |  |  |  |  |  |  |  |  |
| Moderate |  | 1 |  | 6 | 7 | 6 | 5 | 5 | 2 | 1 | 1 | 2 | 1 |  |  |  |  |  |  |  |  |  |
| Moderate to Severe |  |  |  |  | 1 | 2 | 1 |  |  |  |  |  |  |  |  |  |  |  |  |  |  |  |
| Severe |  | 1 | 1 |  |  | 4^†^ | 4 | 5 | 6 | 5 | 2 | 2 | 3 | 3 | 2 | 1 |  |  |  |  |  |  |
| Severe to Very severe |  |  |  |  |  |  |  |  |  |  | 1 | 1 | 1 | 2 | 2 | 1 |  |  |  |  |  |  |
| Very severe |  |  | 1 |  |  | 3^†^ | 3 | 3 | 1 | 4^‡^ | 2^†^ | 2^†^ | 1 | 1^†^ | 2 |  | 1 | 1 | 2 | 1 | 3 | 1 |
| **Reported stool frequency change in relation to the PGIC response options (n)** | | | | | | | | | | | | | | | | | | | | | | |
| Very much better^–^ |  | 1 |  | 1 | 1 | 1 | 1 | 1 | 1 |  | 2 | 1 | 2 | 1 | 2 | 1 |  | 1 |  |  |  | 1 |
| Much better^–^ |  | 1 | 1 |  | 2 | 2 | 1 |  | 1 |  |  |  |  |  | 1 | 1 | 1 | 1 |  |  |  |  |
| A little better to Much better^–^ | 1 |  |  |  |  |  |  |  |  |  |  |  |  |  |  |  |  |  |  |  |  |  |
| A little better^–^ |  |  |  | 1 |  |  | 1 | 1 |  |  |  |  |  |  |  |  |  |  |  |  |  |  |
| No change^–^ |  |  |  |  | 1 |  |  |  |  |  |  |  |  |  |  |  |  |  |  |  |  |  |
| A little worse^+^ |  |  |  |  |  |  |  |  |  |  |  |  |  |  |  |  |  |  |  |  |  |  |
| Much worse^+^ |  |  |  |  |  |  |  |  |  |  |  |  |  |  |  |  |  |  |  |  |  |  |
| Very much worse^+^ |  |  |  |  |  |  |  |  |  |  |  |  |  | 1 |  |  |  |  |  |  |  |  |

† Includes n=1 who stated, ‘or above’, ‡ includes n=3 who stated ‘or above’

– Change corresponds to improvement (reduction in stool frequency); + Change corresponds to worsening (increase in stool frequency).

Darker tone indicates higher row frequency (n). Frequencies do not equate to sample size as some participants provided a range in their responses.
